# Corticosterone accelerates behavioral inflexibility via plasticity-related gene expression in the dorsal striatum

**DOI:** 10.1038/s41386-025-02293-y

**Published:** 2025-12-09

**Authors:** Michael D. Murphy, Keegan S. Krick, Shuo Zhang, Elizabeth A. Heller

**Affiliations:** 1 Department of Systems Pharmacology and Translational Therapeutics, University of Pennsylvania, Philadelphia, PA USA; 2 Penn Epigenetics Institute, Perelman School of Medicine, University of Pennsylvania, Philadelphia, PA USA

**Keywords:** Epigenetics and behaviour, Gene expression, Epigenetics and plasticity, Motivation

## Abstract

Behavioral flexibility allows organisms to modify actions based on new information, such as shifts in reward value or availability, and is promoted by the dorsomedial striatum (DMS). In contrast, behavioral inflexibility provides efficiency and automaticity in familiar contexts, and is promoted by the dorsolateral striatum (DLS). Importantly, chronic elevation of the primary stress hormone, corticosterone (CORT) in rodents or cortisol in humans, impairs behavioral flexibility through dendritic atrophy in the DMS, and promotes inflexible behavioral response strategies through dendritic outgrowth in the DLS. However, understanding of changes in gene expression underlying behavioral inflexibility is lacking. We used a food-motivated operant task in male and female mice to define gene changes that accompany the shift to inflexible behavior with CORT. We discovered that CORT-accelerated loss of behavioral flexibility is accompanied by decreased DMS- and increased DLS-specific synaptic plasticity gene expression, and that distinct genes are either differentially expressed or spliced in the transition to inflexible behavior. Splicing analysis suggests that repressed activity in the DMS during the transition to inflexible behavior may reflect both reduced expression and increased degradation of plasticity-related mRNA transcripts. Finally, given the ability of CORT to influence histone acetylation, we defined CORT-mediated H3K9ac enrichment profiles associated with synaptic plasticity gene regulation stratified by sex and striatal subregion. This study is the first to define CORT-driven epigenetic regulation in the DMS and DLS during the CORT-accelerated transition from flexible to inflexible behavior in male and female mice.

## Introduction

Goal-directed behaviors are motivated by the expectation that actions will result in desirable outcomes. With repetition, such behaviors can take on inflexible, routinized qualities that are insensitive to outcome value [1–3]. Behavioral inflexibility allows organisms to conserve cognitive resources when a given behavior has been reliably reinforced in the past, but this can occur at the expense of adapting to unexpected or negative events. Accordingly, the loss of behavioral flexibility is thought to contribute to psychopathologies, including obsessive-compulsive disorders, addiction, binging disorders, and neurodegenerative diseases [4–9].

The transition from flexible to inflexible behavior naturally occurs with extended experience with a given task and is well characterized and regulated by specific corticostriatal afferents [2, 3]. Most critically, the dorsomedial striatum (DMS) is necessary for producing flexible behavior [1–3, 10, 11], while the dorsolateral striatum (DLS) is a primary driver of inflexible behavior [1–3, 11, 12]. Still, molecular mediators are only beginning to be revealed. The present study aimed to define gene regulation that underlies the transition from behavioral flexibility to inflexibility.

Stress influences decision-making behavior [13, 14]. While acute stress can heighten vigilance and focus, promoting adaptive action [15, 16], chronic stress often impairs cognition, resulting in suboptimal decision-making [15–17] and inflexible response bias [18–20]. The glucocorticoid receptor (GR) is a primary stress hormone receptor in rodents [21–23], activated by the agonist corticosterone (CORT) in rodents and cortisol in humans [24]. Chronic elevated CORT is sufficient to hasten the loss of flexible behaviors in male rodents [18, 25–27]. In this study, we expand these findings to female mice. We quantify treatment and subregion specific gene regulation associated with this transition.

Stress-related changes in decision-making strategies are accompanied by dendritic atrophy on neurons in the DMS and dendritic outgrowth on neurons in the DLS [18]. The structural and morphological adaptations of neurons undergoing synaptic plasticity allow for the refinement of circuit outputs by increasing or decreasing the strength of neuronal connections within or between brain regions [28–30]. We discovered that CORT-accelerated loss of behavioral flexibility is accompanied by synaptic plasticity-related gene expression or alternative splicing in the DMS and DLS. This study is a comprehensive examination of sex- and striatal-subregion-specific gene expression, alternative splicing, and histone acetylation during the loss of behavioral flexibility following extended training or CORT exposure.

## Materials and methods

### Animals

Eight-week-old male and female C57BL/6 J mice were purchased from The Jackson Laboratory (https://www.jax.org/strain/000664) and were housed under a 12-h light-dark cycle (lights on 7:00 AM) at 23 °C. Mice were allowed a week to habituate, then calorie-restricted to maintain 90 ± 5% of their individual baseline body weight for the remainder of the experiments. Mice were housed in same-sex cages with two to four age- and sex-matched cagemates. All experiments were performed in accordance with the University of Pennsylvania Institutional Animal Care and Use Committee and were conducted in accordance with the National Institute of Health Guide for the Care and Use of Laboratory Animals.

### Operant response training

Training began after 2 weeks of water (Vehicle, CORT, or CORT + MIF) administration when mice were 11 weeks old. Mice were habituated to handling for 3 days prior to the initiation of training, and all training sessions were held between 7:30 A.M. and 11:30 A.M. Sessions began by turning on the house light and ventilation fan within each sound-attenuating chamber (Med Associates, MED-307W, Fairfax, VT). Each operant conditioning chamber was designated to one sex and water condition to avoid distracting scents. Mice underwent one session per day, during which pressing a lever initially resulted in a 20 mg sugar pellet reward outcome (Bio-Serv, F07595, Flemington, NJ). Mice progressed to the next training phase when at least 18/20 (90%) of possible reinforcers were earned within the two 40-min sessions. The session would end when a subject earned 20 reinforcers or after 40-min. Food port entries, lever presses, reinforcers earned, and session times were recorded to compute food port entry rates and lever pressing rates.

Operant conditioning proceeded in a fashion similar to prior investigations [31–33]. After training mice according to a fixed ratio 1 (FR1) schedule of reinforcement, described above, mice graduated to a random interval 30s (RI-30s) schedule of reinforcement. Here, responses are not reinforced for randomized periods averaging 30s, after which, the next lever press is reinforced. RI schedules, with time, induce routinized habit-based response strategies because they uncouple the predictive relationship between actions and outcomes. Again, mice were required to accumulate 90% of available pellets to proceed to the next phase of the experiment. After three successful sessions of training according to the RI-30s schedule, mice graduated to an RI-60s schedule. Mice completed either three (limited training) or eight (extended training) sessions of RI-60s training, and the following day proceeded to the devaluation test. For molecular experiments, tissue was collected 1 h after the mouse’s final RI-60s training session.

### Reward devaluation

Each mouse was placed individually in a clean empty cage without bedding, where it had free access to 2 g of the same sugar pellets used as reinforcers for 1.5 h. After freely consuming the reward, mice were transferred to their respective operant conditioning chambers. Lever-pressing was monitored for 10 min in an unrewarded test. Since training responses (food port entries and lever presses) varied based on session timeout, response rates were calculated to inform reinforcer value. Data are expressed as % baseline, referring to the response rate generated during testing compared to the last two RI-60s training days, as previously described in the literature [34–39]. Raw lever pressing rates for the last two RI-60s sessions and the unrewarded test are also shown per mouse. Next, mice were returned to cages containing sucrose pellets for another 1.5 h to repeat the consumption test. The purpose of the consumption test was to provide a measure for each subject’s relative value for the sucrose pellet, with the expectation that after free consumption, the sucrose pellet would be devalued by the mice. Finally, mice were returned to their home cages. Pellet intake was measured by weighing the sugar pellets before and after each consumption session.

### Statistical analysis for behavioral studies

Data are expressed as mean ± standard error of the mean (SEM). Statistical analyses were performed using GraphPad Prism software (version 10.2.2, La Jolla, CA). For all behavioral analyses, we employed an ANOVA followed by a Tukey or Šidák post-hoc test with an alpha of 0.05 to correct for multiple comparisons. Sex as a biological variable was considered in the statistical analyses of all behavioral results. Two-way ANOVA was used to compare two variables (i.e., sex and training), and post-hoc comparisons followed interaction effects. Three-way ANOVA was used to compare three variables (i.e., sex, CORT, and training), with post-hoc comparisons following interaction effects. Full F statistics are reported for ANOVAs, while degrees of freedom (DF) are reported for post-hoc comparisons. The “training day” variable was included as a repeated-measure across all operant conditioning sessions to analyze food port entry rates and lever pressing rates for individual mice undergoing multiple days of operant sessions. The “training duration” variable was included as a discrete variable (limited or extended) when analyzing testing session data, as mice could only receive one pre-training before testing. The Grubbs test was used to identify outliers. Outlier mice were removed from all analyses without replacement. A table of all relevant statistical analyses for each Figure and Supplemental Figure has been provided, with all experimental variables included.

### Tissue collection

Mice were euthanized 1 h after their final RI-60s training session by cervical dislocation. The whole brain was immediately extracted and chilled for a few seconds in phosphate-buffered saline (PBS, pH 7.4) with cOmplete, EDTA-free Protease Inhibitor tablets (Roche, 4693159001, Manheim, Germany) on wet ice. The brain was then placed in a chilled slicer matrix, coronally sectioned at 1 mm increments, and two slices (Fig. S6A, Bregma +0.14 mm A.P. and +1.18 mm A.P. [40]), containing the regions of interest were placed in a sterile dish with chilled PBS + protease inhibitor. Bilateral 2 mm-diameter biopsy punches were taken for each region of interest from two coronal slices (4 total punches per region per animal), placed in individual microcentrifuge tubes, and immediately flash frozen in a metal tube rack placed on dry ice. Samples were stored at −80 °C. Given that the DMS and DLS have some degree of overlap in the central part between the substructures [41, 42], samples potentially contained small amounts of the reciprocal subregion.

### RNA and chromatin isolation for sequencing

The same tissue samples were processed for RNA and chromatin using single sample sequencing (S3EQ) [43–46]. Samples were submitted to Azenta Life Sciences (Plainfield, NJ) for sequencing, where RNA underwent additional quality control assays for RIN (8.5 to 9.9) and RNA concentration (28–98 ng/µL). ChIP DNA underwent tape station (Azenta) and nanodrop (Azenta) analysis to ensure quality before sequencing.

## Results

### Chronic CORT administration accelerates the loss of behavioral flexibility

**Fig. 1:**
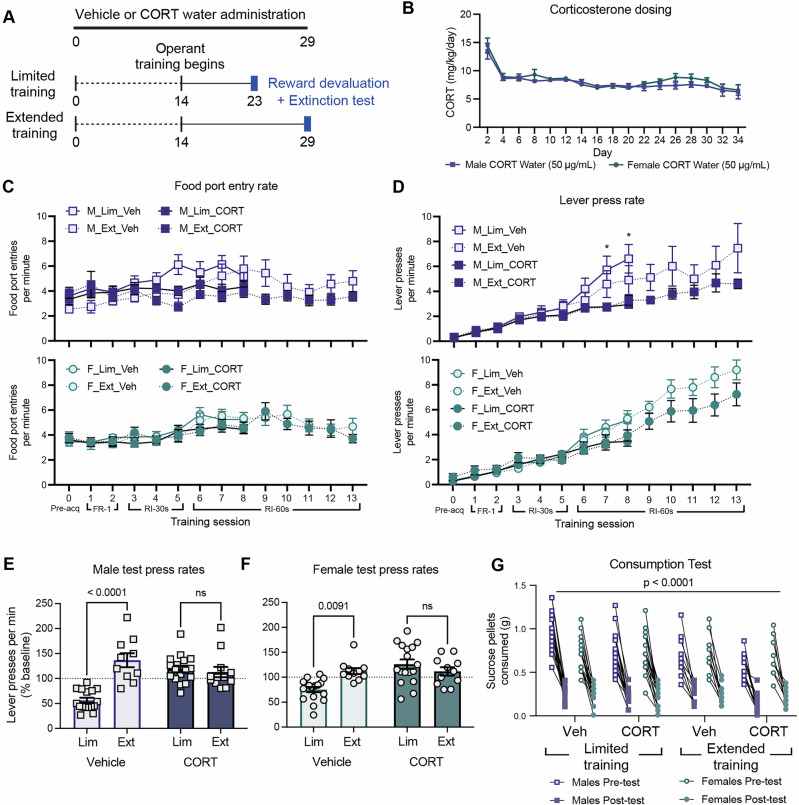
Chronic CORT administration accelerates the loss of behavioral flexibility. **A** Behavioral timeline where CORT or vehicle water administration began 14 days before training and lasted throughout the duration of training and testing. All mice underwent 2 days of FR-1 training, 3 days of RI-30s training, then either 3 days of RI-60s training for limited training groups or 8 days of RI-60s training for extended training groups. Mice progressed to a reward devaluation and testing the day after their final RI-60s training session. **B** There was no sex difference in CORT dosing when adjusting for body weight and water intake per cage [*n* = 12–13 cages/sex, two-way ANOVA, main effect of Time, *F*_(16, 296)_ = 18.36, *p* < 0.0001; main effect of Sex, *F*_(1, 23)_ = 1.842, *p* = 0.1878]. **C, D** The average food port entry rate per minute or lever pressing rate per minute ± SEM by sex (male or female), training duration (limited or extended), and treatment (vehicle or CORT) is shown across each day of the operant conditioning task. CORT had no effect on food port entries in limited [*n* = 16–17 mice/group, three-way ANOVA, main effect of Training Day, *F*_(8, 495)_ = 10.96, *p* < 0.0001; main effect of Sex, *F*_(1, 62)_ = 1.147, *p* = 0.2884; main effect of CORT, *F*_(1, 62)_ = 3.515, *p* = 0.0655] or extended groups [*n* = 10–12 mice/group, three-way ANOVA, main effect of Training Day, *F*_(13, 516)_ = 6.474, *p* < 0.0001; main effect of Sex, *F*_(1, 40)_ = 2.652, *p* = 0.1113; main effect of CORT, *F*_(1, 40)_ = 1.495, *p* = 0.2286]. CORT decreased the lever pressing rates in limited [*n* = 16–17 mice/group, three-way ANOVA, main effect of Training Day, *F*_(8, 423)_ = 76.77, *p* < 0.0001; main effect of Sex, *F*_(1, 53)_ = 0.2827, *p* = 0.5971; main effect of CORT, *F*_(1, 53)_ = 6.999, *p* = 0.0107] and extended groups [*n* = 10–12 mice/group, three-way ANOVA, main effect of Training Day, *F*_(13, 516)_ = 83.37, *p* < 0.0001; main effect of Sex, *F*_(1, 40)_ = 3.321, p = 0.0759; main effect of CORT, *F*_(1, 40)_ = 4.346, *p* = 0.0435]. All mice exhibited an increase in lever pressing as the random interval for training increased. **E**, **F** In an unrewarded lever pressing test following sensory-specific satiety devaluation, vehicle-extended training and both CORT training groups do not attenuate lever pressing and display inflexible lever pressing [*n* = 10–17 mice/group, three-way ANOVA followed by Tukey post hoc tests, main effect of Training Duration, *F*_(1, 52)_ = 0.7872, *p* < 0.0001; main effect of Sex, *F*_(1, 52)_ = 12.85, *p* = 0.987; main effect of CORT, *F*_(1, 52)_ = 38.91, *p* = 0.0002; VehMalesLimited v VehMalesExtended, DF = 103, *p* < 0.0001; VehMalesLimited v CORTMalesLimited, DF = 103, *p* < 0.0001; VehMalesLimited v CORTMalesExtended, DF = 103, *p* < 0.0001; VehFemalesLimited v VehFemalesExtended, DF = 103, *p* = 0.0091; VehFemalesLimited v CORTFemalesLimited, DF = 103, *p* < 0.0001; VehFemalesLimited v CORTFemalesExtended, DF = 103, *p* = 0.0478]. Lever presses per minute from the test are reported as the percent of an individual’s baseline from the average of the individual’s last two completed training sessions. **G** All mice reduced free consumption of the sucrose reward following testing compared to their pre-test consumption during sensory-specific satiety devaluation [*n* = 10–17 mice/group, three-way ANOVA, main effect of Pre v Post Consumption Test, *F*_(1, 107)_ = 594.4, *p* < 0.0001]. See Table 1 for detailed statistics for Fig. 1. See Fig. S1 for detailed plasma CORT levels and Table S1 for subsequent statistics.

Operant conditioning occurs when mice engage in a behavior (e.g., lever pressing) to earn a reinforcement (e.g., sucrose). A limited number of operant training days results in flexible operant behavior, which is sensitive to changes in reinforcement value or availability. Extended training causes motor sequences to become routinized, resulting in inflexible behavior, which is insensitive to changes in reinforcement value [47, 48]. Stress exposure promotes inflexible behavior through activation of the glucocorticoid receptor (GR), a primary factor controlling the stress response [49], and plasma CORT increases with chronic stress [50, 51]. To examine the molecular underpinnings of CORT-accelerated behavioral inflexibility, we trained both male and female mice in a standard protocol for operant conditioning under limited and extended training conditions [31–33], with CORT or vehicle treatment for 2 weeks prior to and throughout training and testing (Figs. 1A, B and S1A–C; Table S1). We assigned mice to either vehicle (1% ethanol) or CORT (50 µg/mL) drinking water groups. The CORT dose was based on previous literature [25, 26, 52] and a dose-response study that confirmed an average CORT dose of 8 mg/kg/day in male and female mice (Fig. 1B; *n* = 12–13 mice/group, two-way ANOVA, main effect of Sex, *F*_(1, 23)_ = 1.842, *p* = 0.1878; Table 1).

**Table 1:** Detailed statistics for Fig. 1.

Figure	Metric	ANOVA comparison	*F*(DFn, DFd)	p value
Fig. 1B	Corticosterone dosing	Time	*F* (16, 296) = 18.36	<0.0001
		Sex	*F* (1, 23) = 1.842	0.1878
		Time x Sex	*F* (16, 296) = 0.5286	0.9313
Fig. 1C	Food port entry rate - limited training	Training day	*F* (8, 495) = 10.96	<0.0001
		Sex	*F* (1, 62) = 1.147	0.2884
		CORT	*F* (1, 62) = 3.515	0.0655
		Training day x Sex	*F* (8, 495) = 1.664	0.1047
		Training day x CORT	*F* (8, 495) = 1.363	0.2102
		Sex x CORT	*F* (1, 62) = 0.3044	0.5831
		Training day x Sex x CORT	*F* (8, 495) = 1.082	0.3744
Fig. 1C	Food port entry rate - limited training	Males: Vehicle v CORT D0 (Šídák post-hoc)	DF = 476	>0.9999
		Males: Vehicle v CORT D1 (Šídák post-hoc)	DF = 476	>0.9999
		Males: Vehicle v CORT D2 (Šídák post-hoc)	DF = 476	>0.9999
		Males: Vehicle v CORT D3 (Šídák post-hoc)	DF = 476	>0.9999
		Males: Vehicle v CORT D4 (Šídák post-hoc)	DF = 476	>0.9999
		Males: Vehicle v CORT D5 (Šídák post-hoc)	DF = 476	0.8445
		Males: Vehicle v CORT D6 (Šídák post-hoc)	DF = 476	>0.9999
		Males: Vehicle v CORT D7 (Šídák post-hoc)	DF = 476	0.9972
		Males: Vehicle v CORT D8 (Šídák post-hoc)	DF = 476	<0.0001
Fig. 1C	Food port entry rate - limited training	Females: Vehicle v CORT D0 (Šídák post-hoc)	DF = 476	>0.9999
		Females: Vehicle v CORT D1 (Šídák post-hoc)	DF = 476	>0.9999
		Females: Vehicle v CORT D2 (Šídák post-hoc)	DF = 476	>0.9999
		Females: Vehicle v CORT D3 (Šídák post-hoc)	DF = 476	>0.9999
		Females: Vehicle v CORT D4 (Šídák post-hoc)	DF = 476	>0.9999
		Females: Vehicle v CORT D5 (Šídák post-hoc)	DF = 476	>0.9999
		Females: Vehicle v CORT D6 (Šídák post-hoc)	DF = 476	>0.9999
		Females: Vehicle v CORT D7 (Šídák post-hoc)	DF = 476	>0.9999
		Females: Vehicle v CORT D8 (Šídák post-hoc)	DF = 476	>0.9999
Fig. 1C	Food port entry rate - extended training	Training day	*F* (13, 516) = 6.474	<0.0001
		Sex	*F* (1, 40) = 2.652	0.1113
		CORT	*F* (1, 40) = 1.495	0.2286
		Training day x Sex	*F* (13, 516) = 1.180	0.2906
		Training day x CORT	*F* (13, 516) = 2.513	0.0024
		Sex x CORT	*F* (1, 40) = 0.2365	0.6294
		Training day x Sex x CORT	*F* (13, 516) = 1.673	0.063
Fig. 1C	Food port entry rate - extended training	Males: Vehicle v CORT D0 (Šídák post-hoc)	DF = 556	>0.9999
		Males: Vehicle v CORT D1 (Šídák post-hoc)	DF = 556	>0.9999
		Males: Vehicle v CORT D2 (Šídák post-hoc)	DF = 556	>0.9999
		Males: Vehicle v CORT D3 (Šídák post-hoc)	DF = 556	>0.9999
		Males: Vehicle v CORT D4 (Šídák post-hoc)	DF = 556	>0.9999
		Males: Vehicle v CORT D5 (Šídák post-hoc)	DF = 556	>0.9999
		Males: Vehicle v CORT D6 (Šídák post-hoc)	DF = 556	>0.9999
		Males: Vehicle v CORT D7 (Šídák post-hoc)	DF = 556	>0.9999
		Males: Vehicle v CORT D8 (Šídák post-hoc)	DF = 556	>0.9999
		Males: Vehicle v CORT D9 (Šídák post-hoc)	DF = 556	0.9999
		Males: Vehicle v CORT D10 (Šídák post-hoc)	DF = 556	>0.9999
		Males: Vehicle v CORT D11 (Šídák post-hoc)	DF = 556	>0.9999
		Males: Vehicle v CORT D12 (Šídák post-hoc)	DF = 556	>0.9999
		Males: Vehicle v CORT D13 (Šídák post-hoc)	DF = 556	>0.9999
Fig. 1C	Food port entry rate - extended training	Females: Vehicle v CORT D0 (Šídák post-hoc)	DF = 556	>0.9999
		Females: Vehicle v CORT D1 (Šídák post-hoc)	DF = 556	>0.9999
		Females: Vehicle v CORT D2 (Šídák post-hoc)	DF = 556	>0.9999
		Females: Vehicle v CORT D3 (Šídák post-hoc)	DF = 556	>0.9999
		Females: Vehicle v CORT D4 (Šídák post-hoc)	DF = 556	>0.9999
		Females: Vehicle v CORT D5 (Šídák post-hoc)	DF = 556	>0.9999
		Females: Vehicle v CORT D6 (Šídák post-hoc)	DF = 556	>0.9999
		Females: Vehicle v CORT D7 (Šídák post-hoc)	DF = 556	>0.9999
		Females: Vehicle v CORT D8 (Šídák post-hoc)	DF = 556	>0.9999
		Females: Vehicle v CORT D9 (Šídák post-hoc)	DF = 556	>0.9999
		Females: Vehicle v CORT D10 (Šídák post-hoc)	DF = 556	>0.9999
		Females: Vehicle v CORT D11 (Šídák post-hoc)	DF = 556	>0.9999
		Females: Vehicle v CORT D12 (Šídák post-hoc)	DF = 556	>0.9999
		Females: Vehicle v CORT D13 (Šídák post-hoc)	DF = 556	>0.9999
Fig. 1D	Lever pressing rate - limited training	Training day	*F* (8, 423) = 76.77	< 0.0001
		Sex	*F* (1, 53) = 0.2827	0.5971
		CORT	*F* (1, 53) = 6.999	0.0107
		Training day x Sex	F (8, 423) = 0.2266	0.986
		Training day x CORT	*F* (8, 423) = 8.977	<0.0001
		Sex x CORT	*F* (1, 53) = 1.362	0.2484
		Training day x Sex x CORT	*F* (8, 423) = 1.217	0.2872
Fig. 1D	Lever pressing rate - limited training	Males: Vehicle v CORT D0 (Šídák post-hoc)	DF = 476	>0.9999
		Males: Vehicle v CORT D1 (Šídák post-hoc)	DF = 476	>0.9999
		Males: Vehicle v CORT D2 (Šídák post-hoc)	DF = 476	>0.9999
		Males: Vehicle v CORT D3 (Šídák post-hoc)	DF = 476	>0.9999
		Males: Vehicle v CORT D4 (Šídák post-hoc)	DF = 476	>0.9999
		Males: Vehicle v CORT D5 (Šídák post-hoc)	DF = 476	>0.9999
		Males: Vehicle v CORT D6 (Šídák post-hoc)	DF = 476	>0.9999
		Males: Vehicle v CORT D7 (Šídák post-hoc)	DF = 476	0.0042
		Males: Vehicle v CORT D8 (Šídák post-hoc)	DF = 476	<0.0001
Fig. 1D	Lever pressing rate - limited training	Females: Vehicle v CORT D0 (Šídák post-hoc)	DF = 476	>0.9999
		Females: Vehicle v CORT D1 (Šídák post-hoc)	DF = 476	>0.9999
		Females: Vehicle v CORT D2 (Šídák post-hoc)	DF = 476	>0.9999
		Females: Vehicle v CORT D3 (Šídák post-hoc)	DF = 476	>0.9999
		Females: Vehicle v CORT D4 (Šídák post-hoc)	DF = 476	>0.9999
		Females: Vehicle v CORT D5 (Šídák post-hoc)	DF = 476	>0.9999
		Females: Vehicle v CORT D6 (Šídák post-hoc)	DF = 476	>0.9999
		Females: Vehicle v CORT D7 (Šídák post-hoc)	DF = 476	>0.9999
		Females: Vehicle v CORT D8 (Šídák post-hoc)	DF = 476	0.6345
Fig. 1D	Lever pressing rate - extended training	Training day	*F* (13, 516) = 83.37	<0.0001
		Sex	*F* (1, 40) = 3.321	0.0759
		CORT	*F* (1, 40) = 4.346	0.0435
		Training day x Sex	F (13, 516) = 4.321	<0.0001
		Training day x CORT	*F* (13, 516) = 4.045	<0.0001
		Sex x CORT	*F* (1, 40) = 0.1337	0.7165
		Training day x Sex x CORT	*F* (13, 516) = 0.3406	0.9853
Fig. 1D	Lever pressing rate - extended training	Males: Vehicle v CORT D0 (Šídák post-hoc)	DF = 556	>0.9999
		Males: Vehicle v CORT D1 (Šídák post-hoc)	DF = 556	>0.9999
		Males: Vehicle v CORT D2 (Šídák post-hoc)	DF = 556	>0.9999
		Males: Vehicle v CORT D3 (Šídák post-hoc)	DF = 556	>0.9999
		Males: Vehicle v CORT D4 (Šídák post-hoc)	DF = 556	>0.9999
		Males: Vehicle v CORT D5 (Šídák post-hoc)	DF = 556	>0.9999
		Males: Vehicle v CORT D6 (Šídák post-hoc)	DF = 556	>0.9999
		Males: Vehicle v CORT D7 (Šídák post-hoc)	DF = 556	>0.9999
		Males: Vehicle v CORT D8 (Šídák post-hoc)	DF = 556	>0.9999
		Males: Vehicle v CORT D9 (Šídák post-hoc)	DF = 556	>0.9999
		Males: Vehicle v CORT D10 (Šídák post-hoc)	DF = 556	>0.9999
		Males: Vehicle v CORT D11 (Šídák post-hoc)	DF = 556	>0.9999
		Males: Vehicle v CORT D12 (Šídák post-hoc)	DF = 556	>0.9999
		Males: Vehicle v CORT D13 (Šídák post-hoc)	DF = 556	0.8447
Fig. 1D	Lever pressing rate - extended training	Females: Vehicle v CORT D0 (Šídák post-hoc)	DF = 556	>0.9999
		Females: Vehicle v CORT D1 (Šídák post-hoc)	DF = 556	>0.9999
		Females: Vehicle v CORT D2 (Šídák post-hoc)	DF = 556	>0.9999
		Females: Vehicle v CORT D3 (Šídák post-hoc)	DF = 556	>0.9999
		Females: Vehicle v CORT D4 (Šídák post-hoc)	DF = 556	>0.9999
		Females: Vehicle v CORT D5 (Šídák post-hoc)	DF = 556	>0.9999
		Females: Vehicle v CORT D6 (Šídák post-hoc)	DF = 556	>0.9999
		Females: Vehicle v CORT D7 (Šídák post-hoc)	DF = 556	>0.9999
		Females: Vehicle v CORT D8 (Šídák post-hoc)	DF = 556	>0.9999
		Females: Vehicle v CORT D9 (Šídák post-hoc)	DF = 556	>0.9999
		Females: Vehicle v CORT D10 (Šídák post-hoc)	DF = 556	>0.9999
		Females: Vehicle v CORT D11 (Šídák post-hoc)	DF = 556	>0.9999
		Females: Vehicle v CORT D12 (Šídák post-hoc)	DF = 556	>0.9999
		Females: Vehicle v CORT D13 (Šídák post-hoc)	DF = 556	>0.9999
Fig. 1E, F	Male and Female test press rates	Training Duration	*F* (1, 52) = 0.7872	<0.0001
		Sex	*F* (1, 52) = 12.85	0.987
		CORT	*F* (1, 52) = 38.91	0.0002
		Training duration x Sex	F (1, 52) = 0.005158	0.0292
		Training duration x CORT	*F* (1, 52) = 0.4073	< 0.0001
		Sex x CORT	*F* (1, 52) = 6.164	0.6316
		Training duration x Sex x CORT	*F* (1, 52) = 9.935	0.1414
Fig. 1E, F	Male and Female test press rates	Veh_Male_Limited training v Veh_Male_Extended training (Tukey post-hoc)	DF = 103	<0.0001
		Veh_Male_Limited training v CORT_Male_Limited training (Tukey post-hoc)	DF = 103	<0.0001
		Veh_Male_Limited training v CORT_Male_Extended training (Tukey post-hoc)	DF = 103	<0.0001
		CORT_Male_Limited training v Veh_Male_Extended training (Tukey post-hoc)	DF = 103	0.8207
		CORT_Male_Limited training v CORT_Male_Extended training (Tukey post-hoc)	DF = 103	0.9995
		Veh_Female_Limited training v Veh_Female_Extended training (Tukey post-hoc)	DF = 103	0.0091
		Veh_Female_Limited training v CORT_Female_Limited training (Tukey post-hoc)	DF = 103	<0.0001
		Veh_Female_Limited training v CORT_Female_Extended training (Tukey post-hoc)	DF = 103	0.0478
		CORT_Female_Limited training v Veh_Female_Extended training (Tukey post-hoc)	DF = 103	0.9333
		CORT_Female_Limited training v CORT_Female_Extended training (Tukey post-hoc)	DF = 103	0.9141
		Veh_Male_Limited training v Veh_Female_Limited training (Tukey post-hoc)	DF = 103	<0.0001
		CORT_Male_Limited training v CORT_Female_Limited training (Tukey post-hoc)	DF = 103	0.997
		Veh_Male_Extended training v Veh_Female_Extended training (Tukey post-hoc)	DF = 103	0.5883
		CORT_Male_Extended training v CORT_Female_Extended training (Tukey post-hoc)	DF = 103	>0.9999
Fig. 1G	Consumption test	Pre v Post	*F* (1, 107) = 594.4	<0.0001
		Sex	*F* (1, 107) = 0.6156	0.4344
		CORT	*F* (1, 107) = 9.567	0.0025
		Pre v Post x Sex	*F* (1, 107) = 2.705	0.103
		Pre v Post x CORT	*F* (1, 107) = 1.677	0.1981
		Sex x CORT	*F* (1, 107) = 6.795	0.2487
		Pre v Post x Sex x CORT	*F* (1, 107) = 2.401	0.1242

Either two-way or three-way analysis of the variance (ANOVA) tests were run with either a Šidák or Tukey post-hoc to correct for multiple comparisons. Corresponding figures, metrics, ANOVA comparisons, F statistics, and *p* values are shown.

We first tested the hypothesis that CORT extinguishes behavioral flexibility in both male [25–27] and female mice. To avoid modeling natural variation in pressing rates, we confirmed that groups did not differ during FR-1, RI-30s, nor through RI-60s day 3. Subjects were assigned to limited or extended training groups based on days 2 and 3 of random interval (RI-)60 s lever pressing rates during (Fig. S2A; Table S2). Food port entry was reduced by CORT in both males and females (Fig. 1C; *n* = 10–17 mice/group, three-way ANOVA, main effect of Training Day, *F*_(13, 1051)_ = 12.29, *p* < 0.0001; main effect of Sex, *F*_(1, 107)_ = 0.1941, *p* = 0.6604; main effect of CORT, *F*_(1, 107)_ = 7.502, *p* = 0.0072; Table 1). Lever pressing increased across training days, as expected. CORT decreased lever pressing rates during training in both male and female mice, compared to vehicle (Fig. 1D; *n* = 10–17 mice/group, three-way ANOVA, main effect of Training Day, *F*_(13, 1051)_ = 132.5, *p* < 0.0001; main effect of CORT, *F*_(1, 107)_ = 22.97, *p* < 0.0001; interaction effect of Training x CORT, *F*_(13, 1051)_ = 9.861, *p* < 0.0001; Table 1), as previously reported with CORT-treated male mice [26]. There was no sex difference in lever pressing rates among vehicle or CORT groups (Fig. 1D; *n* = 10–17 mice/group, three-way ANOVA, main effect of Sex, *F*_(1, 107)_ = 0.855, *p* = 0.3572; interaction effect of Sex x CORT, *F*_(1, 107)_ = 2.17, *p* = 0.1437; Table 1).

Following limited or extended training, mice underwent sucrose reward devaluation through satiety, followed by an unrewarded lever pressing test. It is known that non-devalued limited and extended training male mice do not differ in lever pressing rates during an unrewarded probe test [31, 33, 39], even with chronic stress or CORT treatment [25–27]. However, devalued extended training male mice press more during an unrewarded probe test, indicating a transition to inflexible behavior [25–27, 31, 33, 39]. We therefore hypothesized that limited operant training with CORT would cause insensitivity to reward devaluation and behavioral inflexibility in both male and female mice. We confirmed this with a standard reinforcer devaluation paradigm previously applied only to male rodents. As expected, mice that underwent limited training reduced lever pressing relative to their training baseline, demonstrating sensitivity to reduced reinforcer value. Alternatively, mice that underwent extended training did not integrate reinforcer value into their response strategies, with response rates equivalent to their training baseline (Fig. 1E; *n* = 10–17 mice/group, three-way ANOVA followed by Tukey post-hoc tests, VehMaleLimitedTraining v VehMaleExtendedTraining, DF = 103, *p* < 0.0001; Table 1; Fig. 1F; *n* = 10–17 mice/group, three-way ANOVA followed by Tukey post-hoc tests, VehFemaleLimitedTraining v VehFemaleExtendedTraining, DF = 103, *p* = 0.0131; Table 1; Fig. S3A–H; Table S3). Furthermore, CORT-treated mice with limited training did not exhibit sensitivity to reinforcer value, responding as during training and to the same extent as the extended training groups (Fig. 1E; *n* = 10–17 mice/group, three-way ANOVA followed by Tukey post-hoc tests, VehMaleLimitedTraining v CORTMaleLimitedTraining, DF = 103, *p* < 0.0001; CORTMaleLimitedTraining v VehMaleExtendedTraining, DF = 103, *p* = 0.7106; Table 1; Fig. 1F; *n* = 10–17 mice/group, three-way ANOVA followed by Tukey post-hoc tests, VehFemaleLimitedTraining v CORTFemaleLimitedTraining, DF = 103, *p* < 0.0001; CORTFemaleLimitedTraining v VehFemaleExtendedTraining, DF = 103, *p* = 0.9946; Table 1). Insensitivity to reinforcer value was not due to a failure to devalue the sucrose, as all mice consumed less sucrose in the post-probe consumption test (Fig. 1G; *n* = 10–17 mice/group, three-way ANOVA, main effect of Pre v Post Consumption Test, *F*_(1, 107)_ = 594.4, *p* < 0.0001; Table 1). These differences in testing press rates were also not attributable to differences in baseline training press rates (Fig. S2B, C; Table S2). Finally, we linked CORT treatment to GR by confirming that co-administering mifepristone, a GR antagonist, rescues the CORT-accelerated loss of behavioral flexibilty in both male and female mice (Fig. S4A–H; Table S4). Taken together, we showed that CORT is sufficient to extinguish behavioral flexibility in both male and female mice.

### Dorsal striatum plasticity gene expression signatures during the CORT-accelerated loss of behavioral flexibility

**Fig. 2:**
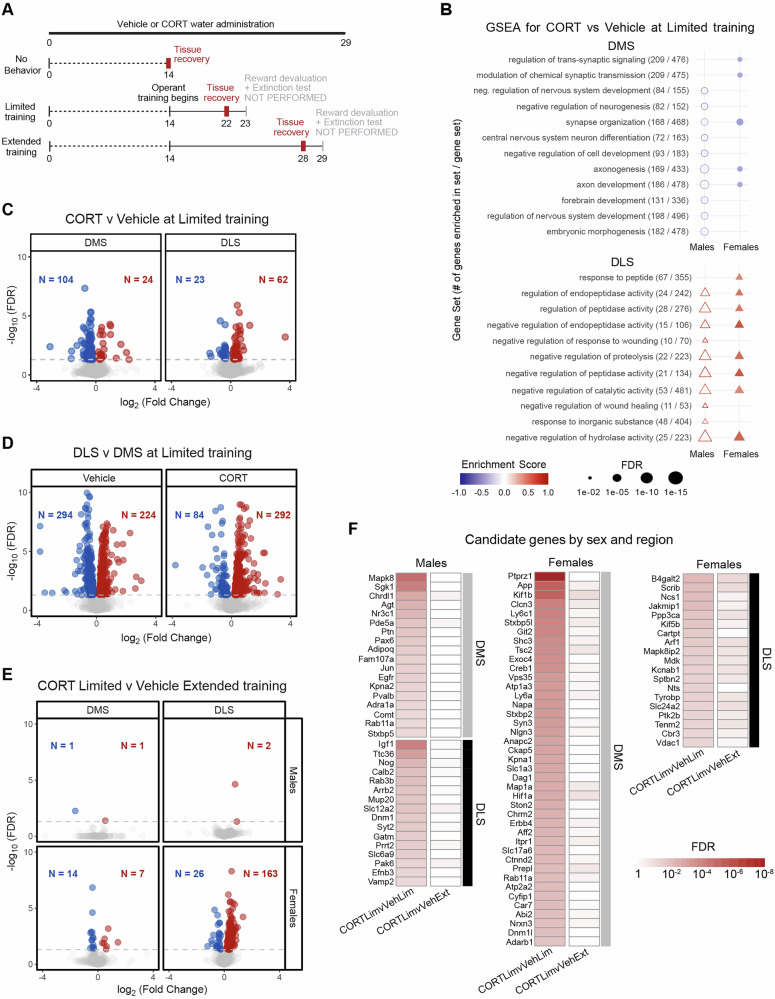
Dorsal striatum plasticity gene expression signatures during the CORT-accelerated loss of behavioral flexibility. **A** Behavioral timeline for tissue recovery from No Behavior, Limited training, and Extended training time points. **B** A gene set enrichment analysis (GSEA) based on all differential gene expression was performed for CORT versus vehicle groups after limited training. An independent GSEA was performed for each sex and brain region combination (*n* = 3/sex/region/treatment/training). The top ten GSEA terms by false discovery rate (FDR; Benjamini-Hochberg adjusted *p*-value) are shown for each combination of sex and brain region. The enrichment score is a scaling of the relative enrichment (red) or de-enrichment (blue) of a gene set term. Selection criteria included the top ten regulation terms per sex and region combination that have an FDR < 0.05. DLS terms were positively enriched for cellular activity regulation terms. DMS terms were negatively enriched for plasticity-related terms. See Fig. S5 for tissue recovery locations and Fig. S6 RNAseq principal component analyses. **C**–**E** Differentially expressed genes (DEGs) across 1157 genes from gene ontology terms: “synaptic signaling” and “cognition.” Significance cutoff (horizontal line at FDR = 0.05). Pearson’s Chi-squared tests are performed for DEGs from each comparison (Tables S6–S8). **C** Differential gene expression from CORT versus vehicle groups produces a decrease in plasticity gene expression in the DMS and an increase in plasticity gene expression in the DLS. See Fig. S10 for detailed volcano plots and Fig. S11 for unique or overlapping DEGs. **D** Differential gene expression from DLS versus DMS groups produces similar amounts of up- and downregulated genes in vehicle groups, and produces less downregulation and more upregulation in CORT-treated groups. See Fig. S12 for detailed volcano plots and Fig. S13 for unique and overlapping DEGs. **E** Differential gene expression from CORT-limited versus vehicle-extended groups produces low differences in male DMS and DLS plasticity gene expression, while producing higher differences in female DMS and DLS plasticity gene expression. See Fig. S15 for detailed volcano plots and Fig. S16 for unique and overlapping DEGs. **F** Candidate genes by sex and region were determined by comparing the p.adjust values from the differential expression (DESeq2) of the CORT-limited training versus vehicle-limited training groups (CORTLimvVehLim) against the false discovery rate (FDR) values from the CORT-limited training versus vehicle-extended training groups (CORTLimvVehExt). Genes that were changed in the CORTLimvVehLim comparison (FDR < 0.05) and were also not changed in the CORTLimvVehExt comparison (FDR > 0.05) are shown.

The dorsomedial striatum (DMS) is necessary for producing flexible behavior [1–3, 10], while the dorsolateral striatum (DLS) is a primary driver of inflexible behavior [1–3, 12]. The role of each brain region in each action strategy is supported by behaviorally relevant changes in synaptic morphology and physiology. We thus hypothesized that plasticity-related gene expression in the DMS and DLS reflects flexible or inflexible behavior, respectively. Having established that CORT accelerates the loss of behavioral flexibility (Fig. 1E, F), we next tested the hypothesis that chronic CORT decreases DMS plasticity gene expression following limited training (Fig. 2A). To test this, we reproduced the operant training paradigm (Fig. S5A–C; Table S5) and performed RNA-sequencing on DMS or DLS collected 1 h after the final RI-60s training session (Figs. 2A and S6A), a time point known to induce plasticity-related gene expression [53]. Sequencing results were quality controlled through principal component analysis (Figs. 2A and S6B–E). To ensure gene expression changes were not a result of differing cell-type proportions, we applied cell-type deconvolution and confirmed consistent cell type proportions across all groups (Fig. S7A–D).

To test our hypothesis on plasticity-related gene expression in the DMS, we applied three bioinformatic approaches to agnostically identify functional terms associated with all DEGs for CORT versus vehicle groups (sex-, brain region-, and training duration-matched): gene set enrichment analysis (GSEA), weighted gene co-expression network analysis (WGCNA), and Mouse Pathway-level Information Extractor Model (MousiPLIER) [54, 55]. GSEA found that CORT decreased enrichment for synapse organization, axon development, and other plasticity-related gene sets in the DMS, but not in the DLS, following limited training compared to vehicle (Fig. 2B). Similarly, WGCNA found enrichment of plasticity terms in the DMS between CORT and vehicle-limited training groups (Fig. S8A–D). WGCNA additionally identified differential enrichment of alternative splicing, metabolic pathways, and glial cell functions in response to CORT and operant conditioning, highlighting non-neuronal adaptations to these stimuli and potentially to behavioral and molecular outputs.

To further test the non-neuronal adaptations to CORT and operant conditioning, we applied MousiPLIER, a machine learning approach to identify latent variables related to immune and inflammatory cell functions [55]. We tested CORT treatment against vehicle groups and identified gene networks implicated in metabolism, intracellular signaling cascades, and alternative splicing that were differentially activated by CORT treatment (Fig. S9). MousiPLIER further identified non-neuronal cell-types, such as microglia, cycling basal cells, and vascular endothelial cells, differentially activated via CORT treatment, confirming that plasticity-related activity is only one component of CORT-accelerated molecular phenomenology, and non-neuronal cell types contribute to molecular changes in the dorsal striatum (Fig. S9). For the purpose of this study, we will predominantly expand on plasticity-related functions. However, there remains a wealth of discovery regarding non-plasticity functional changes and differential activity across non-neuronal cell types.

To deepen our understanding of decreased plasticity-related gene set enrichment in the DMS, we next examined gene expression at the individual gene level. Using an approach orthogonal to GSEA, we defined a list of 1157 plasticity-related genes included in the gene ontology (GO) terms, “synaptic signaling” (GO:0099536/2023-07-25) and “cognition” (GO:0050890/2023-07-25). We selected these GO terms because they are enriched in both our dataset and in a meta-analysis of stress-regulated gene expression across behavioral paradigms [56]. We focus on differential plasticity related gene expression between the DMS and DLS, to complement the known physiological transition from DMS to DLS activity during extended training [57]. We aimed to complement these findings by capturing some of the molecular adaptations of these regions that are known to regulate action strategy.

In CORT versus vehicle groups, we found that chronic CORT downregulated expression of plasticity-related genes following limited training in the DMS (Fig. S10A), in concordance with the GSEA outcomes (Fig. 2B). Next, we measured the expression of plasticity-related genes in the DLS, finding that chronic CORT increased plasticity gene expression in this region compared to vehicle (Figs. 2C; Table S6; and S10B). Specifically, in the DLS, CORT upregulated ~3x more plasticity genes than it downregulated; while in the DMS, CORT downregulated ~4x more plasticity genes than it upregulated (Fig. 2C and Table S6). Additionally, the vast majority of these DEGs were unique by sex and region (Figs. 2C and S11A, B). This ratio of CORT-influenced subregion-specific differential expression in DMS and DLS persisted into extended training (Fig. S10C, D). When comparing DLS versus DMS plasticity gene expression, CORT downregulated plasticity DEGs and upregulated greater DEGs compared to vehicle following limited training (Figs. 2D; Table S7; and S12A, B and Table S7), and these changes persisted through extended training (Fig. S12C, D). The number of unique and overlapping DEGs was somewhat similar by sex and treatment (Figs. 2D; Table S7; and S13A, B). GSEA of these data highlighted subregion-specific or overlapping functional pathways among males or females (Fig. S13C). Together, these analyses support our hypothesis that chronic CORT reduces DMS plasticity gene expression while increasing DLS plasticity gene expression following limited training compared to vehicle groups.

We found that the loss of behavioral flexibility following extended training was recapitulated by CORT treatment following limited training (Fig. 1E, F). Additionally, when comparing vehicle-extended to vehicle-limited training groups, GSEA found reductions in plasticity gene set enrichment in the DMS (Fig. S14A) with greater reduction in plasticity gene expression in the female DMS (Fig. S14B). We thus tested the hypothesis that plasticity gene expression during vehicle-extended training was recapitulated during CORT-limited training in males. Indeed, CORT-limited versus vehicle-extended gene expression in males showed almost no differential gene expression (Figs. 2E; Table S8; and S15A, B). Alternatively, in females, gene expression differed between CORT-limited training and vehicle-extended training and was unique by region (Figs. 2E; Table S8; S15C, D; and S16A, B and Table S8). These results suggest that plasticity-related gene expression during vehicle-extended training is recapitulated by limited training with CORT in male, but not female, mice (Fig. S17A–D).

We next defined a set of high-confidence candidate genes that may be driving the CORT-accelerated loss of behavioral flexibility. We compiled a list of differentially expressed plasticity genes due to CORT by comparing CORT-limited training versus vehicle-limited training groups (CORTLimvVehLim). We then identified which of those plasticity genes were no longer differentially expressed when behavioral flexibility was lost in both groups (CORTLimvVehExt) (Fig. 2F). This analysis provides the first list of candidate genes for CORT-influenced gene regulation in male and female DMS and DLS, which suggests distinct gene expression responses to CORT and training by sex and region.

### Alternative splicing signatures during the loss of behavioral flexibility

**Fig. 3:**
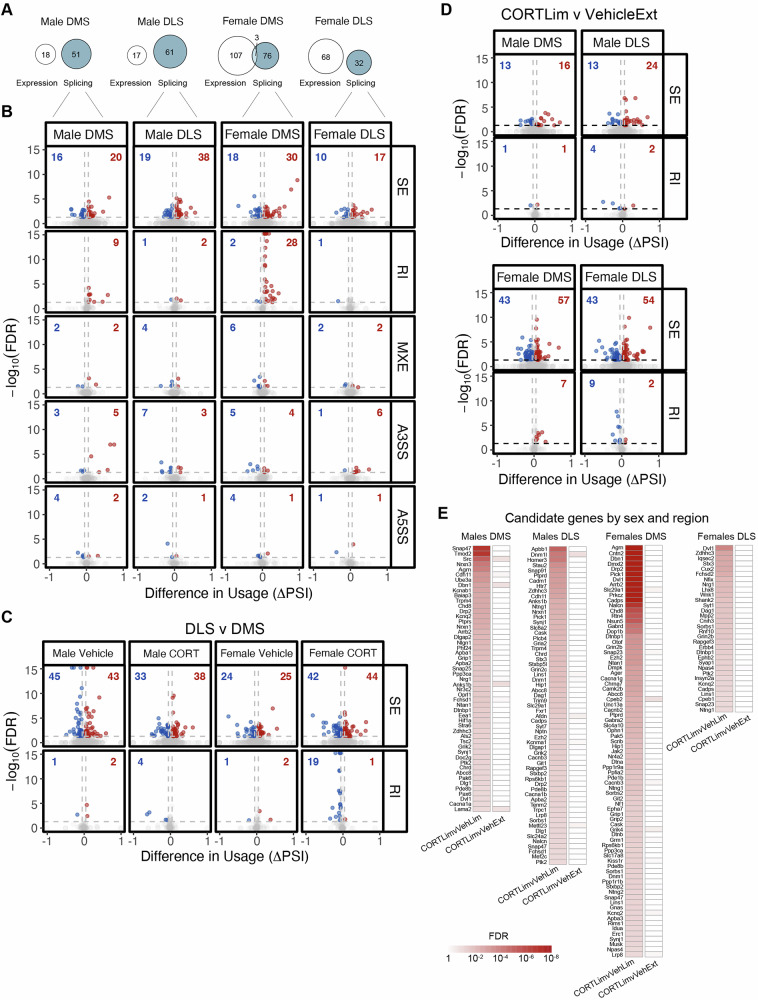
Alternative splicing signatures during the loss of behavioral flexibility. **A** 1157 genes were selected as plasticity-related genes from gene ontology terms: “synaptic signaling” and “cognition”. Of these genes, differential gene expression and differential splice events took place in distinct sets of plasticity genes across sex and region when comparing CORT versus vehicle groups following limited training. **B** CORT administration increased or decreased the number of spliced exons (SEs) inclusions among plasticity genes following limited training. CORT administration increased the number of retained introns (RIs) in the DMS following limited training. There was no established trend for differences in mutually exclusive exons (MXE), alternative 3’ splice site (A3SS), or alternative 5’ splice site (A5SS). See Fig. S18 for relative proportions of SEs, RIs, MXEs, A5SSs, and A3SSs. See Fig. S19 for a gene set enrichment analysis (GSEA) of all differential splice events. **C** Differential inclusion of plasticity gene SEs was the largest difference between DLS and DMS across sex and treatment. The DLS had increased RIs in vehicle females, and the DLS had decreased RIs in male and female CORT groups. **D** There were considerable differences in SEs when comparing CORT-limited training to vehicle-extended groups. Additionally, the female DLS had fewer included RIs, and the female DMS had more included RIs when comparing CORT-limited training to vehicle-extended groups. The male DLS and DMS had relatively low changes in RIs by the same comparison. **E** Candidate genes by sex and region were determined by comparing the false discovery rate (FDR) values from the splice event difference in usage false discovery rate (FDR) of the CORT-limited training versus vehicle-limited training groups (CORTLimvVehLim) against the p.adjust values from the CORT-limited training versus vehicle-extended training groups (CORTLimvVehExt). Genes that were changed in the CORTLimvVehLim comparison (FDR < 0.05) and were also not changed in the CORTLimvVehExt comparison (FDR > 0.05) are shown.

Similar to gene expression, we hypothesized that CORT and training regulated differential alternative splicing in a region-specific manner during the loss of behavioral flexibility. Alternative splicing is an activity-dependent process that regulates transcript isoform abundance and produces functional protein differences such as differential stability, localization, binding properties, or enzymatic activity [58, 59]. Importantly, neuronal activation regulates distinct genes by either differential alternative splicing or differential expression, requiring distinct bioinformatic analyses [45, 60, 61]. We first quantified alternative splicing events in each region, sex, and treatment condition. Next, we measured alternative splicing of plasticity-related genes as defined above. Critically, we found that plasticity-related genes were regulated by either differential expression or alternative splicing across sex and region (Fig. 3A), such that distinct sets of plasticity genes are regulated at the level of total mRNA or the ratio of distinct isoforms.

Given that we observed region and sex-specific alternative splicing regulation of plasticity genes when comparing vehicle-extended training to vehicle-limited training (Fig. S14C), we hypothesized that CORT regulates alternative splicing of plasticity-related genes. We quantified five types of alternative splicing events (skipped exon, SE, retained intron, RI, mutually exclusive exon, MXE, alternative 3′ splice site, A3SS, or alternative 5′ splice site, A5SS) in CORT versus vehicle groups following limited training across. Among plasticity-related genes, alternative SEs exhibited the greatest number of differential splicing events between CORT and vehicle groups following limited training, while the number of differential RI events was particularly distinct among DMS groups (Figs. 3B and S18). Interestingly, GSEA analysis revealed sex- and region-specific term enrichment, and possible cell-type differences, across all alternatively spliced genes (Fig. S19). Comparing sex- and treatment-matched DLS versus DMS groups further suggested SEs were the most commonly disrupted splice event following limited training (Fig. 3C). When comparing DLS versus DMS splice events, CORT treatment also produced a female-DLS-specific decrease (or female-DMS-specific increase) in RIs at the same limited training time point (Fig. 3C). Our analyses suggest that SEs in the DMS and DLS are sensitive to CORT in both sexes, while RIs in the female DMS and DLS are more sensitive to CORT than in males.

Similar to differential expression (Fig. 2E), we hypothesized that alternative splicing of plasticity-related genes during extended training was recapitulated during CORT-limited training. When comparing CORTLimvVehExt training, we found both increased and decreased inclusion SEs in the DMS and DLS (Fig. 3D). There were differential alternative RI events in the DMS and DLS in females, but not males (Fig. 3D). Our analyses demonstrated that CORT, directly and/or indirectly, regulated total transcript levels or alternative splicing of distinct sets of plasticity-related genes following limited training.

We similarly sought to identify high-confidence candidate genes whose alternative splicing was influenced by CORT and may be driving the loss of behavioral flexibility. We used a similar approach to Fig. 2F to identify which plasticity genes were differentially spliced due to CORT at limited training (CORTLimvVehLim) and no longer differentially expressed when comparing behaviorally inflexible groups (CORTLimvVehExt) (Fig. 3E). This analysis provides the first list of candidate genes for direct and/or indirect CORT-influenced alternative splicing in male and female DMS and DLS. We find a greater number of commonly alternatively spliced genes across sex and region in response to CORT and training compared to the uniqueness of the differential expression candidates (Fig. 2E).

### Sex- and region-specific contributions of H3K9ac to gene regulation during the CORT-accelerated loss of behavioral flexibility

**Fig. 4:**
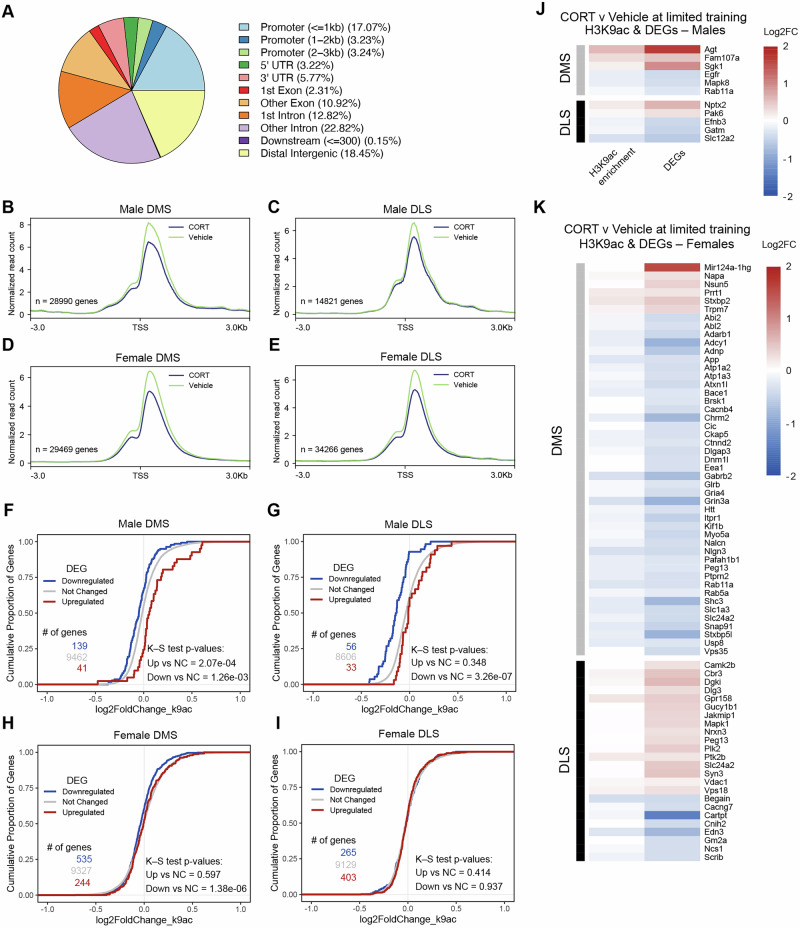
Sex- and region-specific contributions of H3K9ac to gene regulation during the CORT-accelerated loss of behavioral flexibility. **A** H3K9ac enrichment distribution across all sample genomes (*n* = 3/sex/region/treatment/training). **B**–**E** H3K9ac enrichment signal around the transcription start site (TSS). CORT produces a global deenrichment of H3K9ac signal at all gene promoters centered around transcription start sites ± 3 kb for male and female DMS and DLS. **F**–**I** Empirical cumulative distribution function (ECDF) of relative H3K9ac enrichment across the cumulative proportion of genes among differentially expressed genes (DEGs) for male and female DMS and DLS when comparing CORT versus vehicle groups. A leftward shift denotes reduced H3K9ac enrichment, while a rightward shift denotes increased H3K9ac enrichment. Kolmogorov-Smirnov tests are used to test the cumulative H3K9ac enrichment between upregulated (Up) or downregulated (Down) DEGs against genes that were not changed (NC). See Fig. S20 for the additional time points. **J**, **K** H3K9ac enrichment and deenrichment magnitude correlate with unique differentially expressed plasticity genes in male and female DMS and DLS.

Given our findings of CORT-accelerated loss of behavioral flexibility in male and female mice correlating to altered plasticity gene expression, we next examined CORT-mediated chromatin regulation as a potential adaptation at plasticity-related genes. Acute GR activation recruits CREB-binding protein and p300 acetyltransferase to glucocorticoid response elements and increases global histone 3 lysine 9 acetylation (H3K9ac) enrichment in vitro [62, 63], while chronic restraint stress reduces H3K9ac enrichment in mouse hippocampus [64]. Therefore, we hypothesized that GR activation via chronic CORT administration is sufficient to decrease global H3K9ac in mouse DMS and DLS. We measured global H3K9ac by ChIP-sequencing following limited training with CORT or vehicle in the DMS and DLS of the same mice from which we performed RNA-sequencing. We found H3K9ac enrichment in promoter, intragenic, and intergenic regions (Fig. 4A). Given the relevance of promoter H3K9ac enrichment to gene expression, we restricted H3K9ac peak calling to promoter regions ± 3 kb from the transcription start site. Using aggregated H3K9ac promoter signal across all genes with called peaks, we compared CORTLimvVehLim training groups and found that chronic CORT decreased gene promoter H3K9ac enrichment globally compared to vehicle groups (Fig. 4B–E), as expected.

Given our findings of region-specific differences in gene expression due to CORT administration (Fig. 2B–F), we further hypothesized that CORT differentially enriched H3K9ac relevant to differential gene expression, in a brain region-specific manner, following limited training. Differential H3K9ac enrichment analysis was performed between chronic CORT and vehicle groups disaggregated by sex and brain region to obtain log2 fold-change values for H3K9ac enrichment across all differentially expressed genes. In CORT treated tissue, H3K9ac was deenriched at downregulated DEGs in male DMS, male DLS, and female DMS (Fig. 4F–I). H3K9ac was enriched at upregulated DEGs only in the male DMS (Fig. 4F). Sex and region specific correlations between H3K9ac and DEGs were also found in behavior-naive and extended training timepoints (Fig. S20A–H).

We next measured the relationship between CORT-induced differential H3K9ac enrichment and plasticity gene expression. Heatmaps were generated for plasticity DEGs from CORT versus vehicle comparisons that exhibited increased H3K9ac enrichment and increased differential gene expression, or decreased H3K9ac enrichment and decreased differential gene expression. Gene expression and promoter H3K9ac signal of plasticity-related genes following CORT treatment at limited training were unique by sex and subregion (Fig. 4J, K), providing a list of plasticity genes whose expression is potentially regulated by dynamic H3K9ac in response to CORT and training. Taken together, our data supported the hypothesis that differential gene expression positively correlates with H3K9ac in the DMS, suggesting region-specific differences in chromatin regulation due to chronic CORT treatment.

## Discussion

The present study found that CORT accelerates reward value insensitivity after limited operant conditioning in male and female mice, a time point when value-based flexible decision making would normally occur (Fig. S21). We established that CORT is necessary for accelerating the transition to behavioral inflexibility and is reversible by mifepristone. Our findings build on prior studies in male rodents, in which chronic stress or CORT induces a loss of flexible decision-making [13, 18, 19], manifesting as reward value insensitivity [25–27]. CORT-accelerated reward devaluation may reflect diminished positive valence of rewards [27, 65–67]. And indeed, we found that chronic CORT reduced overall reward seeking while interfering with reward devaluation and accelerating the transition to inflexible behavior.

We sought to identify molecular mechanisms implicated in the transition to inflexible behavior. We found that the transition from limited to extended training was sufficient to reduce plasticity-related gene expression in the DMS. Remarkably, we found that CORT and limited training synergized to decrease plasticity-related gene expression in the DMS and increase this in the DLS. Importantly, when comparing both behaviorally inflexible (CORT-limited to vehicle-extended) groups, we find that males exhibit very similar differential expression while females exhibit unique differential expression. Therefore, CORT accelerated the onset of similar plasticity gene expression changes in males, while unique plasticity genes were regulated by the natural or CORT-accelerated shift to inflexible behavior in females. Additionally, our findings support dorsal striatal subregion-specific sensitivity to CORT and resulting activity patterns [68–70], and now contextualizes these as sex-specific as well.

Other groups report similar sex-specific molecular phenomena concurrent with behavioral similarities [71]. For example, CORT-release pellet implantation in mice disrupts reward-seeking behaviors by decreasing dopamine reuptake in male DMS and reducing total dopamine levels in female DMS [71]. Further characterization of candidate genes from our differential expression analysis exemplifies sex-specific cellular and molecular responses to the same stimuli while maintaining expected behavioral outcomes. For instance, in male DMS, there is a decrease in mitogen activated protein kinase 8 (Mapk8), while female DMS exhibits decreased cAMP response element binding protein 1 (Creb1). Mapk8 is a stress activated protein that controls phosphorylation of T-SNARE protein and neurotransmitter vesicle release, which subsequently modulates dopaminergic cell activity [72, 73]. Meanwhile, Creb1 is a transcription factor canonically implicated with learning and memory by promoting neurotrophic factors and modulating synaptic architecture [74]. These differences in neuronal responses contribute to synaptic plasticity through different modalities, but interestingly, the aggregate response across the unique plasticity DEGs still facilitates behavioral inflexibility.

Our MousiPLIER results and candidate genes also highlight the potential for sex- and subregion-specific non-neuronal adaptations that may contribute to CORT-altered plasticity. For instance, male DMS also exhibited decreased chordin-like 1 (Chrdl1) protein, while female DMS exhibited decreased protein tyrosine phosphatase receptor type z1 (Ptprz1). Chrdl1 is released by astrocytes to promote the maturation of neuronal connections via GluA2 AMPA receptors and a net reduction in synaptic kinetics [75]. Meanwhile, Ptprz1 is implicated in astrocyte and oligodendrocyte cell maturation and regulation of myelination[76, 77]. Again, these adaptations were found to be unique by sex and subregion, and highlight unique biological strategies for adaptation to stress and learning among non-neuronal cells in the brain. Future studies should explore this potential for non-neuronal cells and pathways outside of canonical plasticity functions to influence both morphology- and circuit-level neuronal adaptation.

The role of CORT in driving neuronal plasticity relevant to inflexible behavior is supported by previous studies in which stress or CORT increases dendritic density and complexity of DLS neurons [18, 78] and DLS-dependent learning [79, 80]. Alternatively, DMS loss-of-function enhances DLS-guided behavior acquisition [10, 81, 82], and stress reduces dendritic arbor complexity and plasticity in the DMS [18]. Although CORT-limited and vehicle-extended groups both exhibit behavioral inflexibility, it remains to be seen whether the plasticity-related gene expression changes due to CORT-limited or vehicle-extended training directly constitute similar morphological changes in the DMS and DLS. Future studies should interrogate whether these overlapping or unique plasticity gene events constitute similar or unique morphology, dendritic complexity, or circuit-level activity. The current study offers subregion-specific gene-regulatory insights into the canonical transition between DMS- and DLS-guided behavior, in the presence or absence of CORT. One limitation is that our findings reflect the interaction effects of operant learning and chronic CORT on behavioral and molecular outputs. Future studies should additionally evaluate how additional regions that contribute to inflexible behaviors —such as the amygdala [20, 83] and prefrontal cortex [84, 85]—modify their cell-type specific plasticity gene expression in response to CORT or operant conditioning.

Regarding mechanisms by which CORT regulates gene expression, we considered that chronic stress desensitizes neuronal GR and reduces downstream gene expression in the prefrontal cortex and hippocampus [86, 87]. Our chronic CORT treatment also led to a global deenrichment of H3K9ac at DMS and DLS gene promoters following operant conditioning [64]. Our findings of CORT-reduced H3K9ac enrichment may be due to GR desensitization, which would be the first evidence for striatal-specific GR desensitization following chronic CORT. Alternatively, our chronic CORT may not desensitize GR in vivo, and downstream GR signaling from CORT treatment may be sufficient for the molecular changes observed. A limitation of our experimental design was only producing a chronic CORT state; future studies should consider the effects of acute CORT on operant and molecular outputs.

This study is the first to include both male and female mice in a study of CORT-accelerated behavioral inflexibility. We found very few DEGs between CORT-limited training and vehicle-extended training males, suggesting a single set of DEGs to promote inflexible behavior downstream of either extended or CORT-limited training. CORT may bring these gene expression patterns online earlier to produce inflexible decision-making phenotypes. It is also possible that CORT produces unique gene expression, particularly in females, that is not present during the natural shift to inflexible behavior but contributes to accelerated inflexible behaviors. Gene expression in both male and female mice was unique and displayed subregion- and treatment-specific activity patterns that correspond to loss of behavioral flexibility. Yet we found a high number of female-specific CORT-driven DEGs, SEs, and RI events, and correlated H3K9ac/DEG enrichment loci, suggesting that females use overlapping, but distinct, gene expression pathways downstream of extended or CORT-limited training to drive inflexible behavior.

Beyond gene expression, we examined alternative splicing, which regulates >95% of expressed genes in neurons [58, 59]. Critically, activity-dependent alternative splicing profiles are distinct from DEG profiles, yet few studies examine both modes of gene expression [60]. We found that plasticity genes are either differentially expressed or differentially spliced due to chronic CORT. SEs were the most commonly altered splicing event, which can influence transcript diversity and protein function [58]. Additionally, increased RIs in the DMS have the dual capacity to predispose transcripts for degradation, reducing expression [88] or increasing the proportion of nuclear-retained mRNA poised for export and translation under neuronal stimulation [89, 90]. As such, the repressed DMS activity we found during the transition to inflexible behavior may reflect both reduced gene expression and potentially increased degradation of plasticity-related mRNA transcripts due to changes in retained intron proportions. Future studies should consider the molecular impact of GR antagonism as potentially protective against stress- or CORT-induced gene expression changes.

In sum, we discovered that DMS repression and DLS upregulation of synaptic plasticity gene expression accompany the natural and CORT-accelerated loss of behavioral flexibility in male and female mice. Our results support striatal subregion and sex-specific contributions to flexible decision-making via differential gene expression, alternative splicing, and permissive chromatin. Future endeavors should consider sex- and region-specific profiling of GR transcription factor binding or other chromatin modifications that may contribute to chronic stress-potentiated gene dysregulation and behavioral outcomes.

## Supplementary information

Supplemental Materials

Table S2

Table S3

Table S4

Table S5

Table S8

Dataset 1

Dataset 2

## Data Availability

The datasets used and analysed during the current study are available on Gene Expression Omnibus (GEO#: GSE312440), and code is available on GitHub (https://github.com/HellerLAbeats). Additional data are available from the corresponding author on reasonable request. Detailed methods are described in the supplemental materials.
